# Prognostic role of microRNA-203 in various carcinomas: evidence from a meta-analysis involving 13 studies

**DOI:** 10.1186/s40064-016-3225-y

**Published:** 2016-09-13

**Authors:** Ying Liang, Wenhui Yang, Yanhui Zhu, Yulin Yuan

**Affiliations:** 1 Department of Clinical Laboratory, General Hospital of the Yangtze River Shipping, Wuhan, Hubei China; 2 Department of Clinical Laboratory, The People’s Hospital of Guangxi Zhuang Autonomous Region, Nanning, Guangxi China

**Keywords:** MicroRNA-203, Prognosis, Overall survival, Cancer

## Abstract

Growing evidence from recent studies has revealed that microRNA-203 (miR-203) might be an attractive prognostic biomarker for cancer. But controversy still remains. The aim of this meta-analysis was to summarize available evidences and clarify the preliminary predictive value of miR-203 for prognosis in cancer patients. Eligible studies were identified through multiple research strategies in PubMed, EMBASE and Web of Science up to October 2015. Key statistics such as pooled hazard ratios (HR) with 95 % confidence intervals (CIs) were utilized to calculate patient survival. 13 eligible studies with 1600 patients were ultimately enrolled in this meta-analysis. Our results failed to show a significant relation between upregulated miR-203 expression and a favorable overall survival (OS) (HR 1.00, 95 % CI 0.65–1.36) in a random effect model. However, in subgroup analysis, we found that high expression of miR-203 was significantly associated with poor OS in Caucasian patients (HR 1.31, 95 % CI 1.06–1.55). In contrast, for Asian patients, over-expression of miR-203 was an independent prognostic factor for better and OS (HR 0.59, 95 % CI 0.22–0.96). It also suggested that cancer types and miRNA assay method were significant associated with prognosis. The over-expression of miR-203 was effectively predictive of worse prognosis in breast cancer (HR 6.35, 95 % CI 1.34–11.36), pancreatic cancer (HR 1.19, 95 % CI 1.08–1.30), ependymoma (HR 1.35, 95 % CI 1.10–1.61), but for glioma patients, elevated miR-203 is a potential biomarker for predicting better progression of cancer (HR 0.26, 95 % CI −0.02 to 0.54). Besides, for direct miRNA profiling studies, over-expression of miR-203 was an independent prognostic factor for worse OS (HR 6.35, 95 % CI 1.34–11.36). This meta-analysis indicated that ethnicity, tumor type and miRNA assay method mainly contributed to heterogeneity. Considering the insufficient evidence, further relevant studies are warranted.

## Background

MicroRNAs (miRNAs) represent a class of non-coding, highly conserved single stranded RNAs of approximately 22 nucleotides (Fabian and Sonenberg 2012; Guo et al. 2016; Bartel 2004; Carthew and Sontheimer 2009). These tiny regulators mainly function via post-transcriptionally regulating gene expression at the posttranscriptional level (He and Hannon 2004; Garzon et al. 2006; Rolle et al. 2016). They are thought modulate protein production by binding to complementary sites of target messenger RNAs (mRNAs), leading to degradation or transcriptional gene silencing of mRNAs (Filipowicz et al. 2008). In light of previous reports that most human miRNAs are located at cancer associated regions of the genome, it can be speculated that miRNAs may be extensively involved in cancers (Calin et al. 2005; Macfarlane and Murphy 2010; Jonckheere et al. 2015). In recent years, profiling studies have confirmed that miRNAs are aberrantly expressed in various human cancers and are implicated in tumor progression and metastasis (Manikandan et al. 2008; Lu et al. 2005; Cheng 2015).

Mir-203, located at the chromosome 14q32.33, is one of the most frequently mentioned miRNAs. Previous studies have showed that miR-203 exhibited aberrant expression in multiple malignancies compared with their normal tissue, including gastric cancer (Imaoka et al. 2015), pancreatic adenocarcinoma (Ikenaga et al. 2010) and colon carcinoma (Schetter et al. 2008). Many studies have revealed that miR-203 plays an essential role during cell growth, migration, invasion (Viticchie et al. 2011; Wang et al. 2013a). Studies also demonstrate that miR-203 acts as a tumor suppressor in tumor progression (Wang et al. 2014). Therefore, it is hypothesized that miR-203 might be a potential prognostic markers in carcinoma.

Although various studies have focused on the prognostic role of miR-203 in cancer, the conclusion remained inconsistent. In the current study, we carried out a meta-analysis to evaluate and predict the overall risk of elevated miR-203 expression for survival in patients with cancer. In addition, we also explored the implications and feasibility of utilizing miRNA-203 as a prognostic marker in clinical practice.

## Methods

We performed this meta-analysis following the guidelines of the Meta-analysis of Observational Studies in Epidemiology group (MOOSE) (Stroup et al. 2000).

### Search strategy and study selection

We systematically searched online databases, including PubMed (http://www.ncbi.nlm.nih.gov/pubmed/), EMBASE (http://store.elsevier.com/embase) and Web of Science (www.isiknowledge.com), to identify relevant studies published before 6 October 2013. The search strategy included the following combinations of the keywords: ‘microRNA 203’, ‘cancer OR carcinoma OR tumor OR malignancy’ and ‘prognosis OR survival OR mortality OR death OR relapse OR recurrence OR outcome’. The titles, abstracts, full texts and reference lists of retrieved articles were carefully scanned to identify additional eligible studies.

Eligible studies had to meet the following criteria: (1) English publications; (2) a focus on patients with any type of carcinoma; (3) the association between miR-203 expression and survival outcome. Other exclusion criteria included studies were: (1) review articles or letters; (2) duplicate publications; (3) lacked sufficient data.

### Data extraction and quality assessment

For each included study, the following data elements was collected: trial features (e.g., the first author and publication year); characteristics of the participants (e.g., ethnicity, pathological type, sample category, mean age and gender ratio); detection method; follow-up time and data needed for prognosis meta-analysis [e.g., hazard ratios (HR), overall survival (OS) along with their 95 % CIs and P values]. An HR of >1 was considered significant association with increasing risk of mortality or recurrence. If only Kaplan–Meier curves were reported, the data were extracted from graphical survival plots and HR was estimated using previously described methods (Williamson et al. 2002; Tierney et al. 2007). The methodological quality of all the studies included was assessed according a critical review checklist of the Dutch Cochrane Centre proposed by MOOSE (Stroup et al. 2000).

### Statistical analysis

HR and 95 % confidence interval (CI) were conducted as Tierney’s method (Tierney et al. 2007). A test of heterogeneity for pooled HR was carried out by Cochran’s Q test and Higgins I-squared statistic. If heterogeneity was observed (P < 0.05), a random effect model (Der Simonian and Laird method) was adopted, while the fixed effect model was applied in the absence of between-study heterogeneity (P > 0.05) (Higgins et al. 2003). In addition, we also evaluated the publication bias by Egger linear regression test using the funnel plot (Egger et al. 1997). All analyses were conducted with STATA version 12.0 (StataCorp LP, College Station, Texas, USA).

## Results

### Study characteristics

**Fig. 1:**
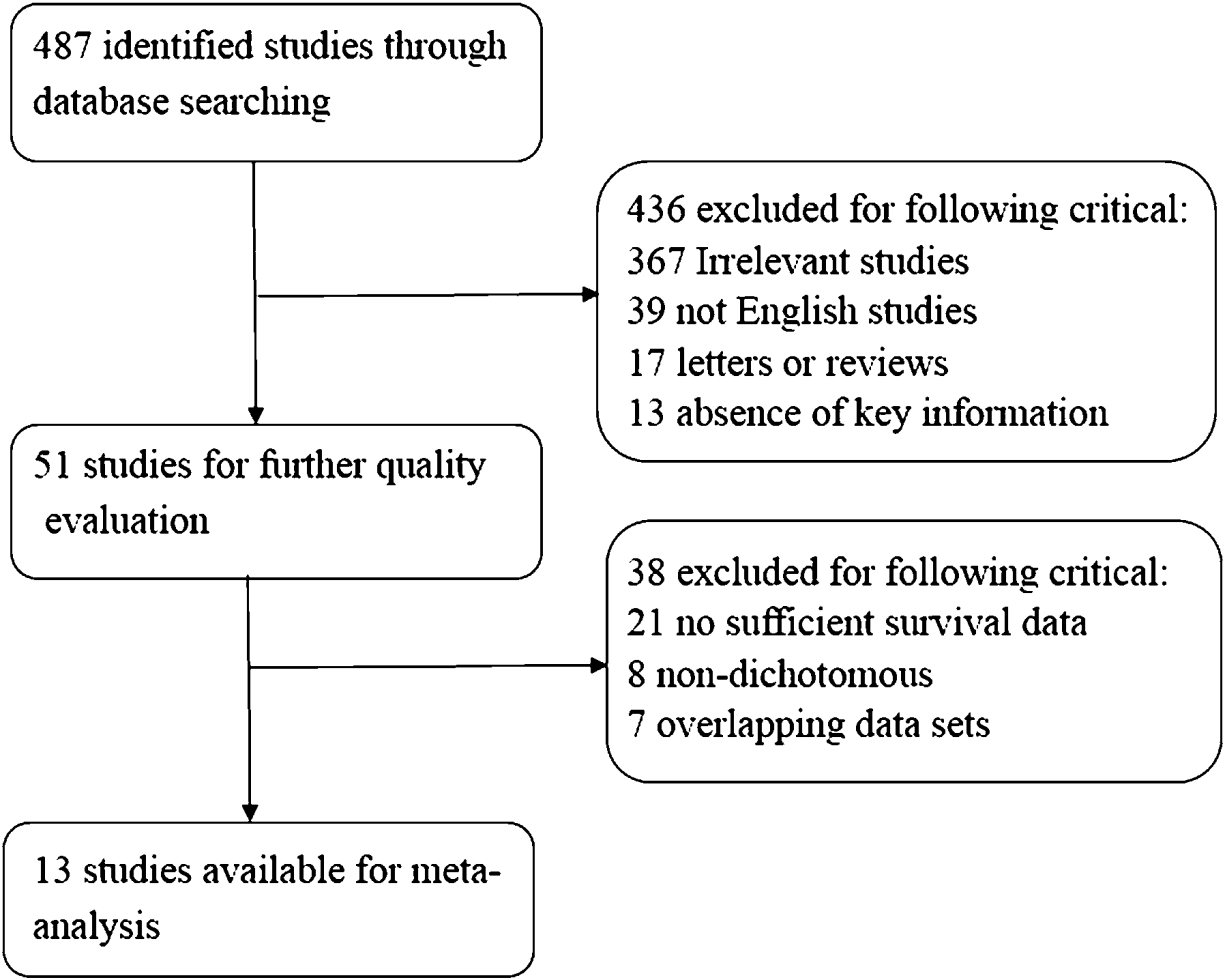
Flow diagram of the study selection process

A total of 487 studies focusing on the relationship between miR-203 and multiple cancers were identified in this meta-analysis. The selection process of candidate studies was shown in Fig. 1. Finally, 13 studies were considered eligible for the analysis, among the 13 included studies, 3 that evaluated pancreatic cancer (Ikenaga et al.^2010^; Greither et al. 2010; Schultz et al. 2012), 2 that evaluated hepatocellular carcinoma (Chen et al. 2012; Liu et al. 2015), colorectal cancer (Bovell et al. 2013; Schetter et al. 2008) and one each that evaluated glioma(He et al. 2013), ovarian cancer (Wang et al. 2013a, b), breast cancer (Madhavan et al. 2012), gastric cancer (Toiyama et al. 2015), esophageal carcinoma (Mathe et al. 2009) and ependymoma (Costa et al. 2011). The main characteristics of these studies are reported in Table 1.

**Table 1:** Characteristics of studies included in the meta-analysis

First author	Years	Country	Gender Male/female	Median age (years)	Disease	Number	Stage I–II/III–IV	miR-203 assay	Source of HR	Maximum months of follow-up
Chen	2012	China	56/10	56 (40–73)	Hepatocellular	66	59/7	qRT-PCR	Reported	100
Liu	2015	China	75/20	52 (29–82)	Hepatocellular	95	22/73	qRT-PCR	Survival curves	68
He	2013	China	70/42	44 (6–86)	Glioma	112	38/74	qRT-PCR	Survival curves	161
Bovell	2013	USA	170/175	<65 169; ≥ 65 176	Colorectal	345	184/161	qRT-PCR	Reported	348
Schetter	2008	USA	66/18	64.6 (32–87)	Colorectal	84	37/46	qRT-PCR	Reported	141.9
Wang	2013	China	NR	50.2 (30–70)	Ovarian	156	48/108	qRT-PCR	Survival curves	88
Madhavan	2012	Germany	NR	61 (30–92)	Breast	133	104/26	Profiling	Survival curves	10.8
Imaoka	2015	Japan	91/39	68	Gastric	130	69/61	qRT-PCR	Reported	78
Ikenaga	2010	Japan	71/42	66 (36–86)	Pancreatic	113	109/4	qRT-PCR	Survival curves	98
Greither	2010	Germany	NR	NR	Pancreatic	50	NR	qRT-PCR	Survival curves	NR
Nicolai	2012	Denmark	111/114	64 (31–85)	Pancreatic	225	35/180	qRT-PCR	Reported	196
Costa	2011	USA	13/21	NR	Ependymoma	34	22/12	qRT-PCR	Reported	160.8
Mathé a	2009	USA	12/12	<62 11; ≥ 62 13	Esophagus	24	21/3	qRT-PCR	Survival curves	NR
Mathé b	2009	Japan	30/3	<62 14; ≥ 62 19	Esophagus	33	19/14	qRT-PCR	Reported	NR

*NR* not reported, *qRT-PCR* quantitative reverse transcription PCR

### Meta-analysis

**Fig. 2:**
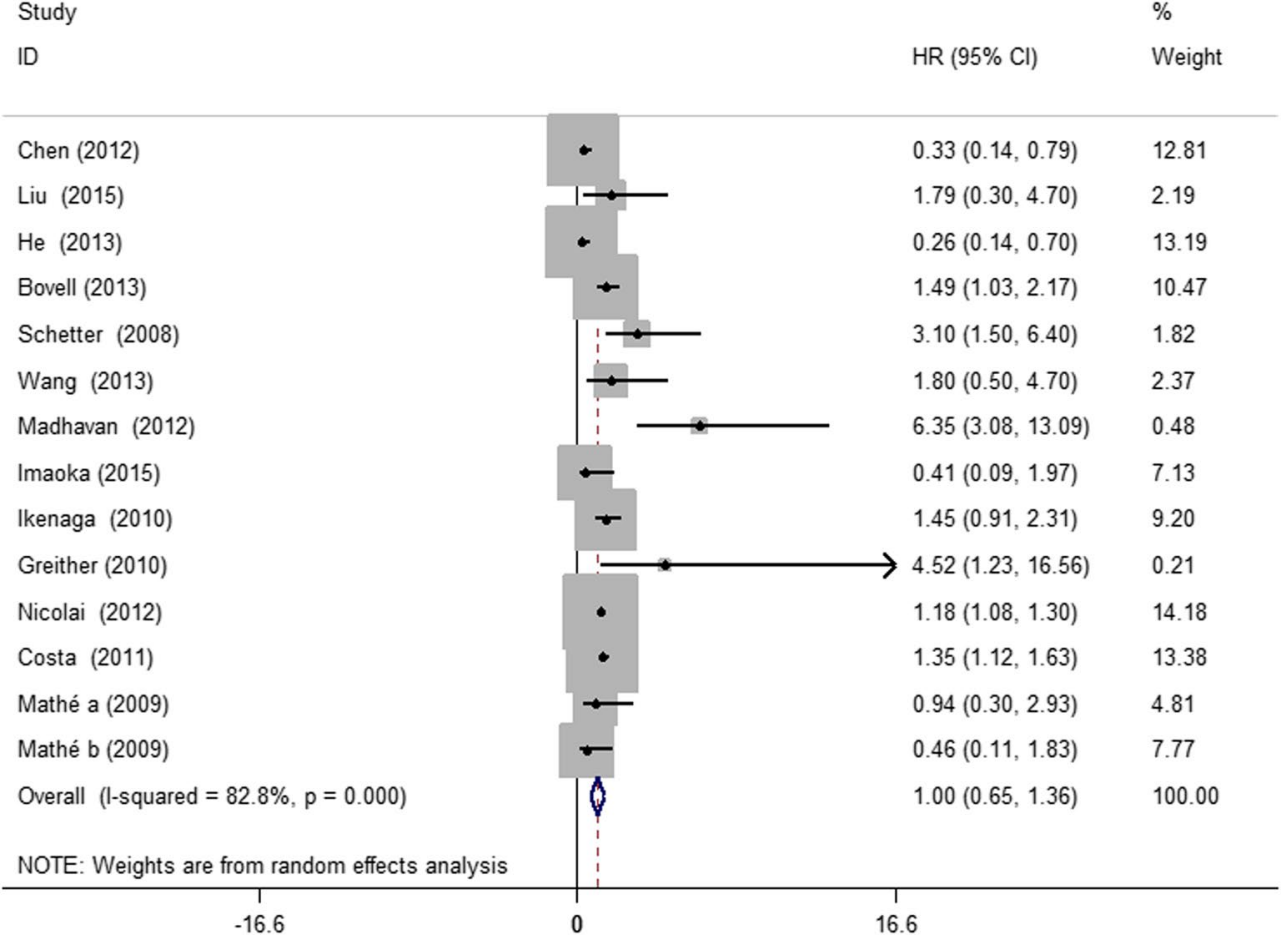
Forest plots of the analyses about miR-203 and overall survival with tumors

The data of 1600 patients with multiple cancers from USA, China, Germany, Japan, Denmark among the included studies was summarized for overall survival (OS) analysis. Since significant heterogeneity among the studies was observed (I^2^ = 82.8 %, P = 0.000), the random effect model was applied to calculate the pooled HR and its 95 % CI. Figure 2 displayed the forest plot of the analysis about miR-203 and OS. The results showed that higher expression levels of miR-203 was not a significant predictor of poor OS, with the pooled HR being 1.00 (95 % CI 0.65–1.36, P = 0.000).

**Fig. 3:**
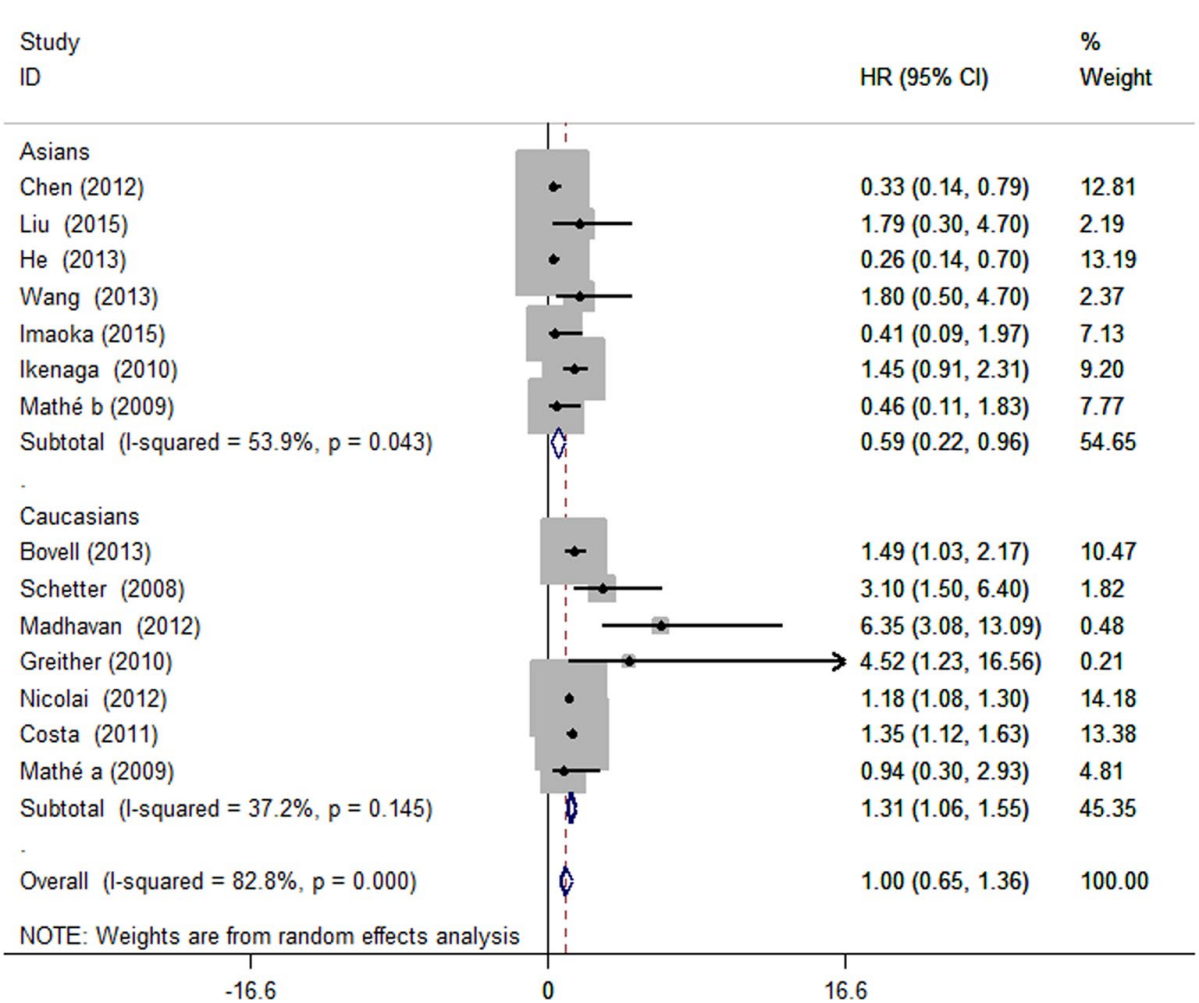
Forest plots derived from the analyses of Caucasians and Asians studies

**Fig. 4:**
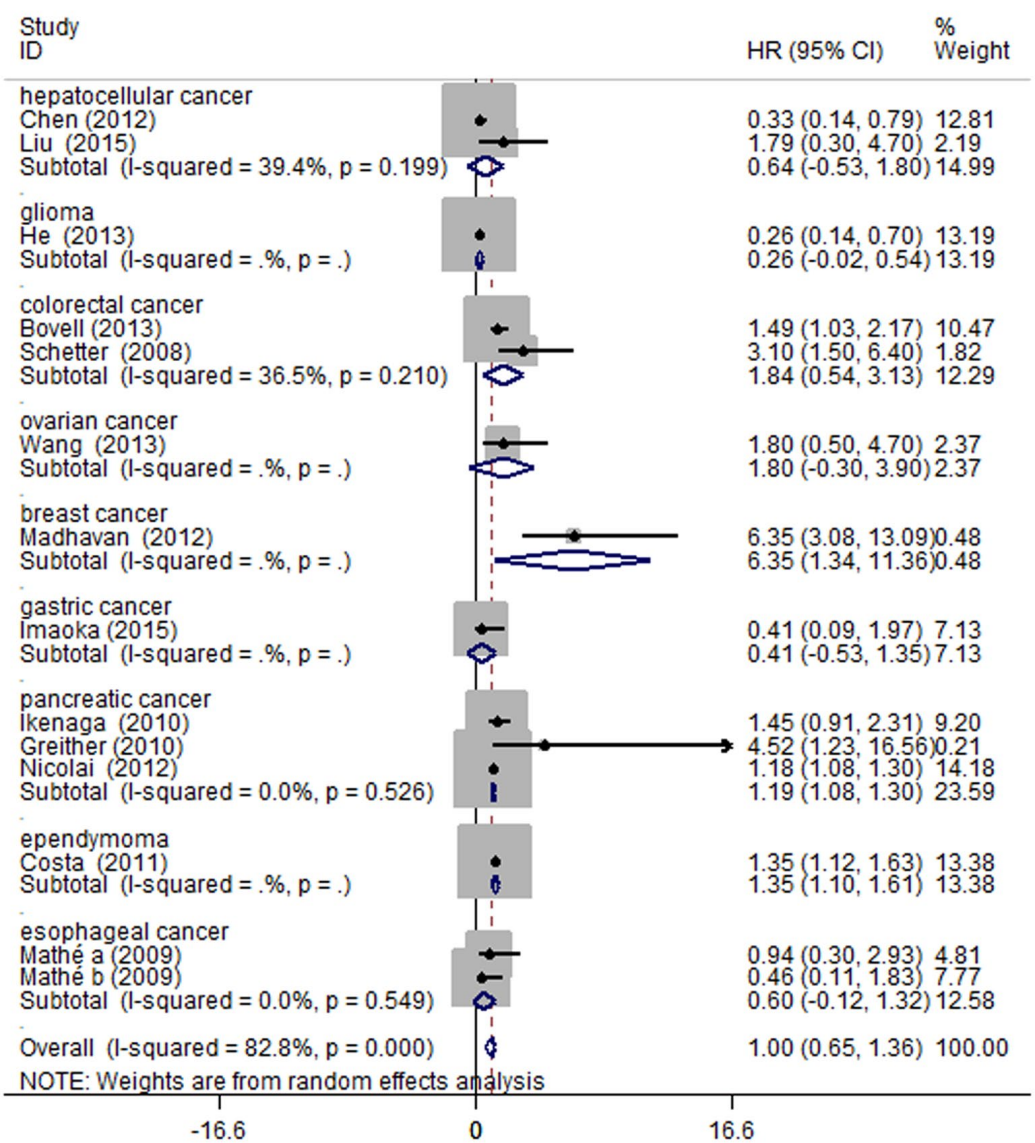
Forest plots derived from the analyses of various cancers

**Fig. 5:**
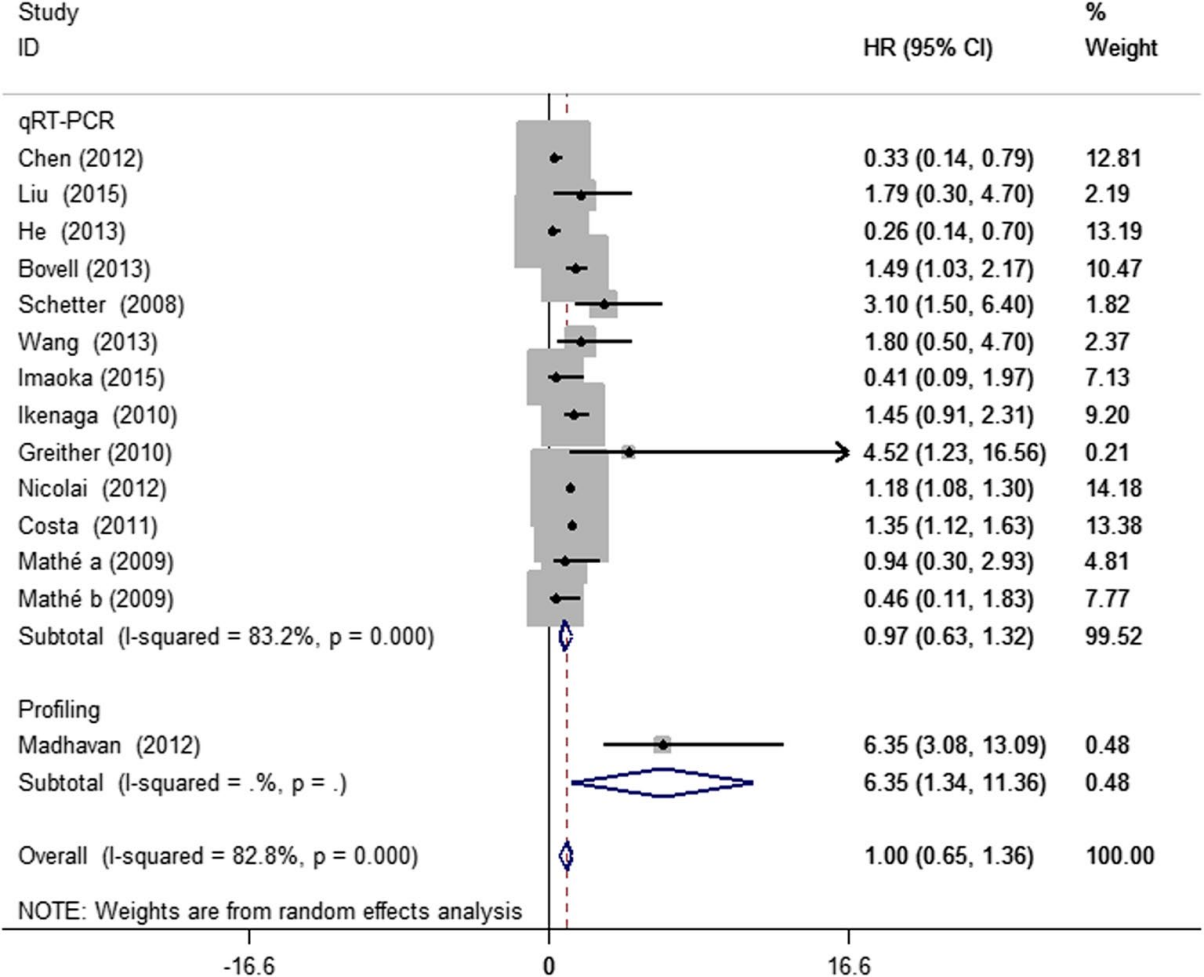
Forest plots derived from the analyses of different assay methods

Afterwards we performed subgroup analyses by classifying each study according to the ethnicity, tumor type and miRNA assay method. For the 7 studies focused on Asians, high miR-203 expression was found to be significantly associated with good OS (HR 0.59, 95 % CI 0.22–0.96), but for the Caucasians, miR-203 high expression was demonstrated to moderately predict poor OS (HR 1.31, 95 % CI 1.06–1.55) (Fig. 3). When grouped according to cancer style of individual studies, as Fig. 4 showed, the combined HR of breast cancer, pancreatic cancer and ependymoma were 6.35 (95 % CI 1.34–11.36), 1.19 (95 % CI 1.08–1.30) and 1.35 (95 % CI 1.10–1.61), respectively, indicating that miR-203 was indicators of poor prognosis in breast cancer, pancreatic cancer and ependymoma. As for glioma, over-expression of miR-203 was predictive of better outcome with the HR of 0.26 (95 % CI −0.02 to 0.54). However, no statistically significant associations were found in hepatocellular carcinoma, colorectal cancer, ovarian cancer, gastric cancer and esophageal carcinoma. In the subgroup analysis by miRNA expression assay method (Fig. 5), there existed significant associations between over-expression of miR-203 and poor prognosis in direct miRNA profiling studies (HR 6.35, 95 % CI 1.34–11.36), but no statistically significant associations were found in qRT-PCR studies. These subgroup analyses results indicated that ethnicity, cancer style and miRNA assay method might be the major source of heterogeneity. Over-expression of miR-203 in Asians was predictive of better outcome, but for Caucasians, it moderately predicted poor OS. For breast cancer, pancreatic cancer and ependymoma, high expression of miR-203 was significantly associated with poor OS. For direct miRNA profiling studies, over-expression of miR-203 was an independent prognostic factor for worse OS.

### Publication bias

**Fig. 6:**
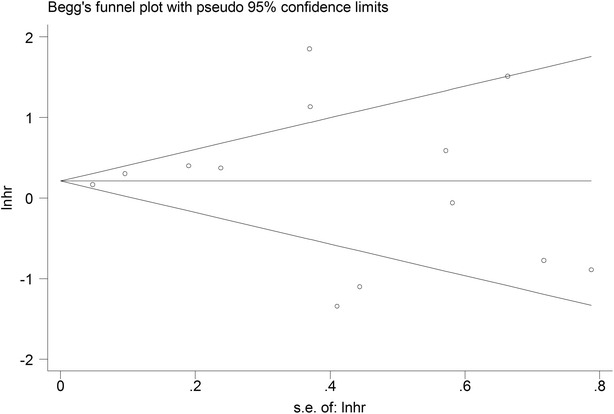
Funnel plots provided graphic estimate of bias for overall studies

Finally, funnel plot and Egger’s test were performed to assess the publication bias of the included literature. As summarized in Fig. 6, the funnel plots were symmetrical well, meanwhile, the P values of the Egger test were 0.804 for OS, indicating publication bias was not detected in the overall analysis of 13 enrolled studies.

## Discussion

Recent studies have demonstrated the aberrant expression of miRNAs in tumors, and specific miRNAs have been found to plays crucial roles in cell growth, migration, invasion and tumor progression (Viticchie et al. 2011; Wang et al. 2013a, 2014). Compare to proteins and mRNAs, miRNAs are more stable and degrade slower, which can be detected as stable markers in serum and even in both formalin-fixed and paraffin-embedded tissues. Therefore, miRNAs attract great attention to be an effective predictor for early diagnosis and accurate prognosis in cancers.

Among these miRNAs, miR-203 has been frequently studied to serve as a tumor suppressor or a tumor promoter in different human cancers. A lot of literatures have confirmed that miR-203 contributes to frequent tumor-specific CpG-island methylation and is closely related to various tumor-associated protein, such as ΔNp 63, Akt 2 and ABL 1 to regulate tumor cell proliferation (Furuta et al. 2010; Yuan et al. 2011; Bueno et al. 2008; Li et al. 2011; Liang et al. 2015). This indicates the important relationship between miR-203 expression and tumor patient survival. Recently, several studies have observed that significant relationship between elevated miR-203 expression and poor overall survival (Schetter et al. 2008; Bovell et al. 2013) in colorectal cancer. Greither et al. (2010) also reported that high expression of miR-203 predicted poor prognosis in pancreatic tumors. However, a recent study published by He’s et al. (2013) concluded that overall survival of patients with high miR-203 expression was significantly longer than those with low miR-203 expression. As we know, no meta-analysis has focused on the potential prognostic role of miR-203 in cancer prognosis. Thus, this meta-analysis was carried out to evaluate the prognostic value of miR-203 in various cancer.

The current meta-analysis has collected 13 articles of 1600 patients. In random effect model, the analysis results of forest plot showed that miR-203 might not have sufficient power to predict the overall survival of cancers, with the pooled HR being 1.00 (95 % CI 0.65–1.36). In order to explore potential sources of heterogeneity, subgroup analyses were performed according to ethnicity, cancer types and miRNA assay method. The subgroup analysis results suggested that high expression of miR-203 was significantly associated with poor OS of cancer patients among Caucasians people (HR 1.31, 95 % CI 1.06–1.55), but in Asians, a better OS associated with elevated miR-203 expression (HR 0.59, 95 % CI 0.22–0.96). Based on the analysis results, ethnicity seems to play a crucial role in association of miR-203 expression and cancer patient prognosis, which mainly due to the variations in genetic background, environmental factors. It was also summarized that cancer types have a considerable impact on the prognostic role of miR-203. High expression of miR-203 moderately predicted poor OS in breast cancer, pancreatic cancer and ependymoma patients, but for glioma patients, high miR-203 expression was significantly associated with better OS. Besides, over-expression of miR-203 was an independent prognostic factor for worse OS when direct miRNA profiling assay method was used. Generally speaking, our results show that miR-203 may not be sufficient to predict cancer prognosis and the ethnicity, cancer types and miRNA assay method may be the main source of discrepancies.

Nonetheless several limitations exist in the present meta-analysis study. First, more and more attention are focused on prognostic role of miR-203 in cancer, but the studies we included are all from Asians and Caucasians, the cancer styles are not comprehensive too; hence, further studies explored the prognostic value of miR-203 on various ethnicities and cancer styles may be needed. Secondly, the numerical value of pooled HR was not sufficiently strong to indicate the prognosis although we have demonstrated the correlation of high miR-203 expression with the overall survival in Caucasians and Asians. Thirdly, we extracted the data form survival curves if the studies didn’t provide HR, which might be of less credibility compared with direct analysis on HR. Despite of the limitations, our study is the first meta-analysis to evaluate the prognostic role of miR-203 in cancer.

## Conclusion

In summary, our findings showed that miR-203 may not be sufficiently accurate as a prognosis for human cancer. But we explored that elevated miR-203 expression might potentially predict the poor survival in Caucasian people while was significantly correlated with good overall survivals in Asians. Besides, tumor type and miRNA assay method may be the potential sources of heterogeneity. More clinical investigations with larger sample size should be conducted to focus on the relationship between miR-203 expression and patient prognosis.
